# In silico analysis of *BRCA1* and *BRCA2* missense variants and the relevance in molecular genetic testing

**DOI:** 10.1038/s41598-021-88586-w

**Published:** 2021-05-27

**Authors:** Kok-Siong Poon

**Affiliations:** Department of Laboratory Medicine, National University Hospital, NUH Main Building, 5 Lower Kent Ridge Road, Singapore, 119074 Singapore

**Keywords:** Cancer, Genetics

## Abstract

Over the years since the genetic testing of *BRCA1* and *BRCA2* has been conducted for research and later introduced into clinical practice, a high number of missense variants have been reported in the literature and deposited in public databases. Polymorphism Phenotyping v2 (PolyPhen-2) and Sorting Intolerant from Tolerant (SIFT) are two widely applied bioinformatics tools used to assess the functional impacts of missense variants. A total of 2605 *BRCA1* and 4763 *BRCA2* variants from the ClinVar database were analysed with PolyPhen2 and SIFT. When SIFT was evaluated alongside PolyPhen-2 HumDiv and HumVar, it had shown top performance in terms of negative predictive value (NPV) (100%) and sensitivity (100%) for ClinVar classified benign and pathogenic *BRCA1* variants. Both SIFT and PolyPhen-2 HumDiv achieved 100% NPV and 100% sensitivity in prediction of pathogenicity of the *BRCA2* variants. Agreement was achieved in prediction outcomes from the three tested approaches in 55.04% and 68.97% of the variants of unknown significance (VUS) for *BRCA1* and *BRCA2*, respectively. The performances of PolyPhen-2 and SIFT in predicting functional impacts varied across the two genes. Due to lack of high concordance in prediction outcomes among the two tested algorithms, their usefulness in classifying the pathogenicity of VUS identified through molecular testing of *BRCA1* and *BRCA2* is hence limited in the clinical setting.

## Introduction

The *Homo sapiens BReast CAncer 1*, *early onset* (*BRCA1*) and *BReast CAncer 2*, *early onset* (*BRCA2*) genes were identified in 1994^1^ and 1995^2^, respectively. They are the two main susceptibility genes which confer high risks in their pathogenic variant carriers for the hereditary breast and ovarian cancers (HBOC). In addition to the *BRCA1* and *BRCA2* genes, there are other susceptibility genes reported to date as high penetrance and moderate penetrance genes involving in the development of breast cancer. The high penetrance genes include the *TP53*^3^, *PTEN*^4^, *STK11*^5^ and *CDH1*^6^. Not only conferring high risk to breast cancer, mutation carriers of the above-mentioned genes are also having higher chances for developing other types of cancerous syndrome with different tiers of risk. On the other hand, moderate penetrance genes such as *PALB2*^7^, *CHEK2*^8^, *ATM*^9^ and *RAD51C*^10^ are also well-studied for their conferred risks to breast cancer. Among of all the afore-mentioned susceptibility genes, *BRCA1* and *BRCA2* remain the two most frequently tested genes for the genetic testing of hereditary breast cancer in the current clinical settings.

The *BRCA1* gene (NCBI RefSeq NG_005905; LRG_292) is located at the chromosome 17q21 and spanning a genomic region of 193 kilo bases. It has several isoforms of messenger RNA (mRNA) transcript. Current standard mRNA transcript, NM_007294 contains 22 coding exons resulting in a translated protein with 1863 amino acids (NP_009225). The *BRCA2* gene (NCBI RefSeq NG_012772; LRG_293) with chromosomal position at 13q13 has a genomic sequence of 91 kilo bases. The standard mRNA transcript is NM_000059. This transcript encodes 3418 amino acids (NP_000050) in 26 coding exons. Both *BRCA1* and *BRCA2* play roles in homologous recombination pathway for repairing double-strand DNA. They are tumor-suppressor genes, hence the mutated alleles give predisposition of cancers to the carriers^11^.

Over the years since the genetic testing of *BRCA1* and *BRCA2* has been conducted for research and clinical practice, a high number of sequence variants have been reported in the literature and deposited in public databases. The sequence variants identified from the studied subjects can be categorized into five classes^12^. Common benign polymorphisms including variants without pathogenicity or of no clinical significance are grouped as Class 1. Class 2 includes variants that are likely benign or of little significance. Class 3 represents variant of unknown significance (VUS) while classes 4 and 5 include likely pathogenic and definite pathogenic variants, respectively. The ENIGMA (Evidence-based Network for the Interpretation of Germline Mutant Alleles) consortium has been playing an important role to evaluate the clinical significance of sequence variants in high-risk breast cancer genes including *BRCA1* and *BRCA2* genes since its establishment in 2009^13^. The BRCA Exchange portal also serves as a consolidated resource for aggregating curated *BRCA1* and *BRCA2* variants for public access^14^.

The frequency of reporting VUS is up to 20% for individuals undergoing genetic testing of *BRCA1* and *BRCA2* but can be lower in other well-studied populations^15^. Identification of VUS is considered as inconclusive and clinically not actionable. While creating uncertainty for treatment decision, reporting VUS has the potential to raise anxiety in its carriers^16^. A large number of VUS have been listed in publicly available databases such as the Breast Cancer Information Core (BIC) database^17^ and ClinVar^18^. The VUS usually consist of non-truncating single nucleotide changes resulting in missense variants, small in-frame insertions or deletions and variants in intronic, non-coding and untranslated regions. The VUS can be novel at the time it was reported due to the unavailability of prior clinical or functional evidence about its causality. Over the time, the status of a particular VUS can be shifted to either benign or pathogenic after sufficient findings have been accumulated^19^.

With the advances of bioinformatics and computational biology, many in silico tools have been developed to help the biologists, scientists, geneticists and clinicians to predict the effects and potential significance of the missense coding variants. Polymorphism Phenotyping v2 (PolyPhen-2)^20^ and Sorting Intolerant from Tolerant (SIFT)^21^ are two widely applied bioinformatics tools used to assess functional effects of missense variants. The PolyPhen-2 gives predictions based on both sequence alignment and structural features characterizing the amino acid substitution while SIFT solely considers the sequence homology and protein conversation among species to assess functional effects of the variants. Apart from that, many other bioinformatics programs based on different algorithms, have been developed. Most of the online tools grant free access to users.

The aim of the present study was to perform in silico analysis for the *BRCA1* and *BRCA2* missense variants which are currently reported in ClinVar including pathogenic variants, benign variants and VUS, using the PolyPhen2 and SIFT.

## Results

### Prediction results from PolyPhen-2 and SIFT on known benign and pathogenic variants

There were 165 and 142 missense variants with known clinical significance (classified as benign or pathogenic), for *BRCA1* and *BRCA2* respectively which were subjected to both PolyPhen-2 and SIFT predictions. The prediction results for the benign and pathogenic entries were summarised in Supplementary Table S1 and Supplementary Table S2 for *BRCA1* and *BRCA2* respectively. For PolyPhen-2, the variants predicted as ‘‘possibly damaging’’ and ‘‘probably damaging’’ were grouped together as “damaging” variants. The positive predictive value (PPV), negative predictive value (NPV), sensitivity, specificity, and accuracy were summarised in Table 1.

**Table 1:** Calculations of positive predictive value (PPV), negative predictive value (NPV), sensitivity, specificity, and accuracy for *BRCA1* (N = 131 Benign, N = 34 Pathogenic) and *BRCA2* (N = 129 Benign, N = 13 Pathogenic).

Gene	PolyPhen-2										SIFT				
	HumDiv					HumVar					PPV	NPV (%)	Sensitivity	Specificity	Accuracy
	PPV	NPV	Sensitivity	Specificity	Accuracy	PPV	NPV	Sensitivity	Specificity	Accuracy					
*BRCA1*	32.61% (28.00% to 37.58%)	94.52% (87.14% to 97.77%)	88.24% (72.55% to 96.70%)	52.67% (43.77% to 61.45%)	60.00% (52.10% to 67.54%)	33.85% (26.52% to 42.03%)	88.00% (82.08% to 92.15%)	64.71% (46.49% to 80.25%)	67.18% (58.43% to 75.12%)	66.67% (58.92% to 73.80%)	32.38% (29.03% to 35.92%)	100	100% (89.72% to 100.00%)	45.80% (37.07% to 54.73%)	56.97% (49.04% to 64.64%)
*BRCA2*	19.12% (16.21% to 22.41)	100%	100% (75.29% to 100.00%)	57.36% (48.36% to 66.03%)	61.27% (52.74% to 69.32%)	27.91% (21.52% to 35.33%)	98.99% (93.70% to 99.85%)	92.31% (63.97% to 99.81%)	75.97% (67.66% to 83.05%)	77.46% (69.70% to 84.05%)	17.11% (14.74% to 19.76%)	100	100% (75.29% to 100.00%)	51.16% (42.21% to 60.06%)	55.63% (47.07% to 63.96%)

### Evaluation of prediction results of variants with known clinical significance

**Figure 1:**
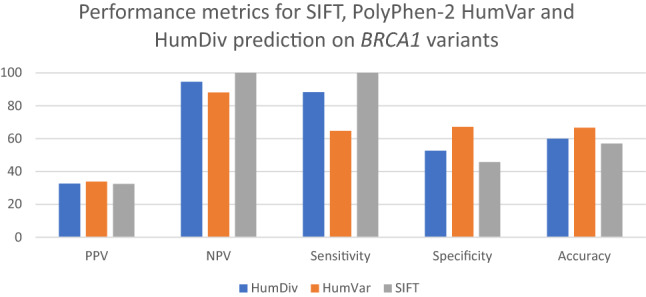
Performance metrics for SIFT, PolyPhen-2 HumVar and HumDiv predictions on *BRCA1* variants with known clinical significance.

**Figure 2:**
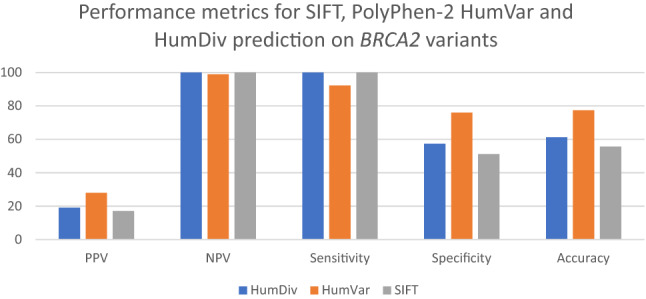
Performance metrics for SIFT, PolyPhen-2 HumVar and HumDiv predictions on *BRCA2* variants with known clinical significance.

Poor performances of PPV were noted for the *BRCA1* and *BRCA2* genes with the top-performers being PolyPhen-2 HumVar—33.85% for *BRCA1* and 27.91% for *BRCA2*, respectively. In terms of NPV, all three approaches achieved good results (NPV > 85%) in the two genes. For *BRCA1* (Fig. 1), SIFT and PolyPhen-2 HumVar showed highest sensitivity (100%) and highest specificity (67.18%), respectively. For *BRCA2* (Fig. 2), both Polyphen-2 HumDiv and SIFT showed 100% sensitivity while Polyphen-2 HumVar achieved highest specificity (75.97%). PolyPhen-2 HumVar showed highest accuracies for *BRCA1* (66.67%) and *BRCA2* (77.46%) genes, respectively.

### Prediction results from PolyPhen-2 and SIFT on *BRCA1* and *BRCA2* missense VUS

**Figure 3:**
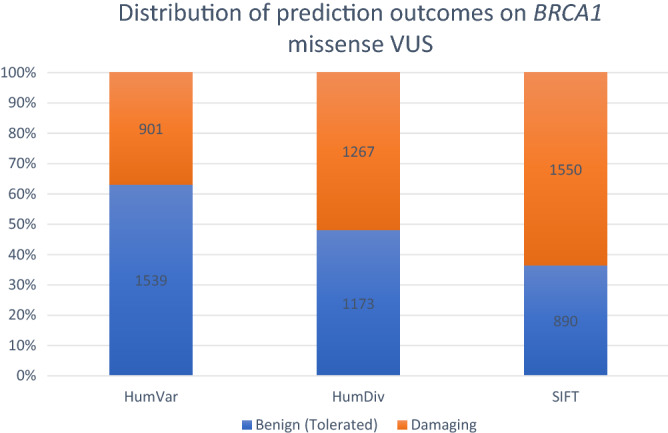
Summary of distribution of prediction outcomes on *BRCA1* missense VUS.

**Figure 4:**
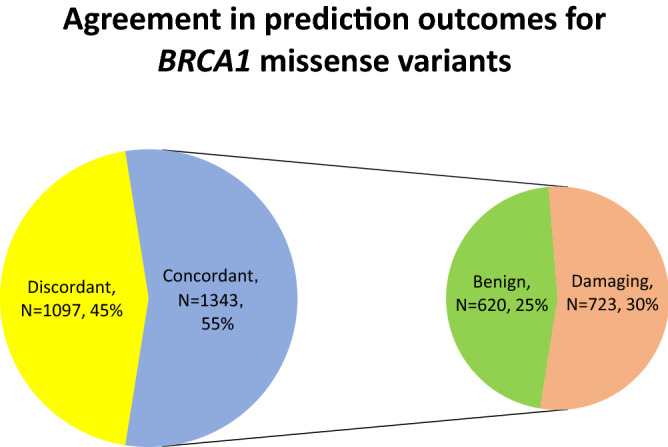
Summary of agreement in prediction outcomes for *BRCA1* missense VUS.

**Figure 5:**
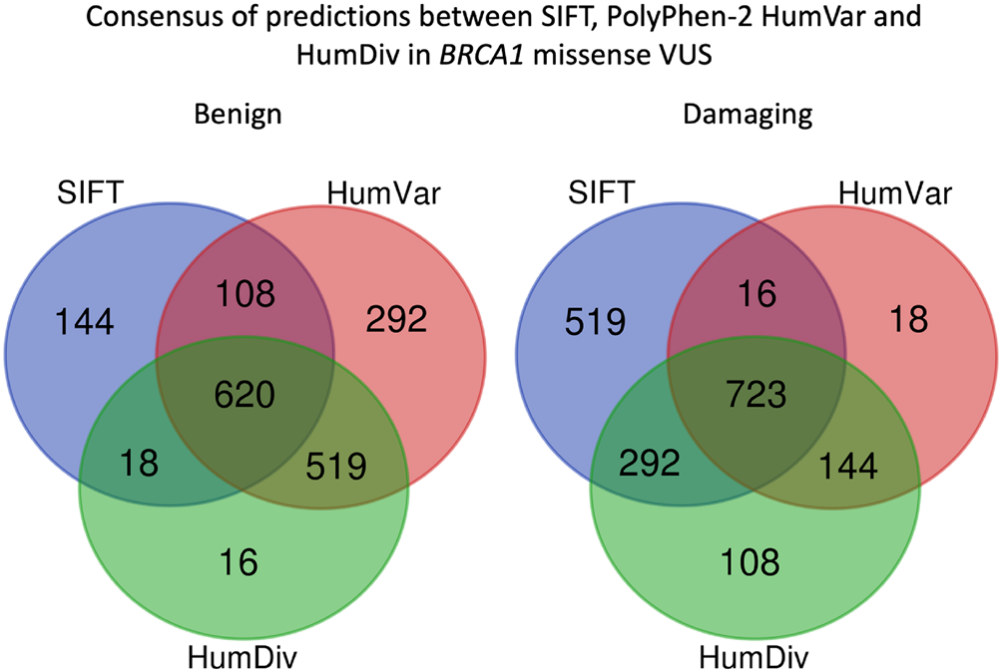
Summary of consensus in prediction outcomes for *BRCA1* missense VUS.

There were 2440 *BRCA1* and 4621 *BRCA2* missense VUS analysed by both PolyPhen-2 and SIFT. For *BRCA1* (Fig. 3), PolyPhen-2 HumDiv predicted benign effects in 48.07% (N = 1173) and damaging effects in 51.93% tested VUS (N = 1267). PolyPhen-2 HumVar predicted benign effects in 63.07% (N = 1539) and damaging effects in 36.93% tested VUS (N = 901). SIFT predicted tolerated effects in 36.48% VUS (N = 890) and damaging effects in 63.52% VUS (N = 1550). Agreement was achieved in prediction outcomes from the three tested approaches in 55.04% (N = 1343) of the tested VUS with 620 and 723 VUS being predicted as benign and damaging, respectively (Fig. 4). On the other hand, PolyPhen-2 HumDiv achieved highest consensus with PolyPhen-2 HumVar in predicting benign VUS. However, for the pathogenic predictions, highest consensus was achieved between SIFT and PolyPhen-2 HumDiv (Fig. 5). Notably, about 26.32% (N = 452) and 35.44% (N = 645) of predictions observed were unique (outstanding predictions given by only one among three algorithm) for benign and damaging categories, respectively. This implied that higher concordance (consensus in at least two out of three tested approaches) was achieved for benign prediction in *BRCA1* VUS.

**Figure 6:**
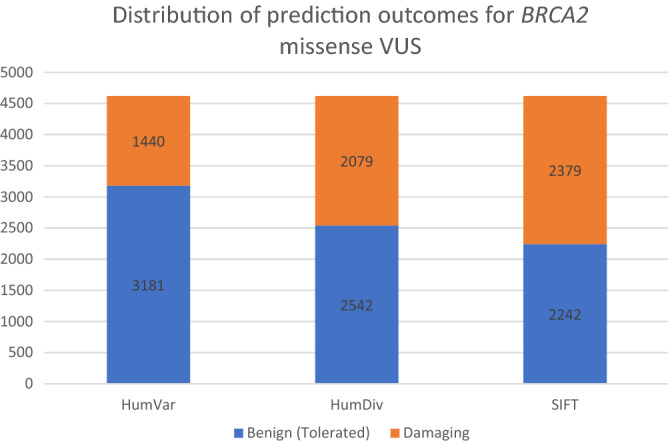
Summary of distribution of prediction outcomes on *BRCA2* missense VUS.

**Figure 7:**
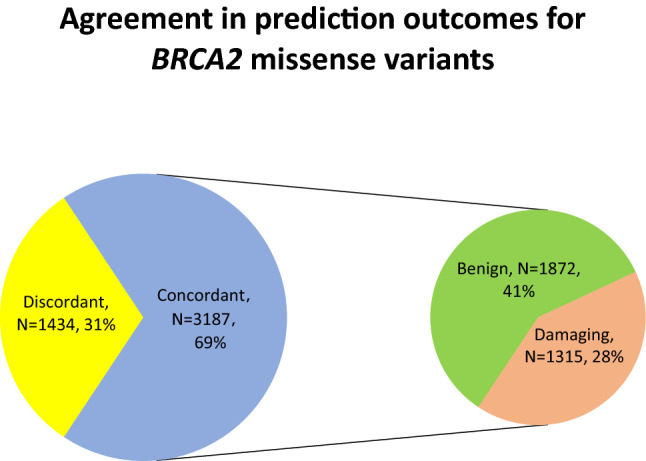
Summary of agreement in prediction outcomes for *BRCA2* missense VUS.

**Figure 8:**
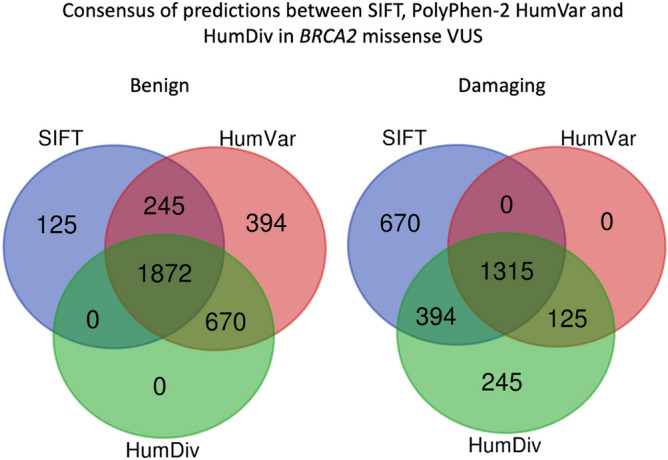
Summary of consensus in prediction outcomes for *BRCA2* missense VUS.

On the other hand, for *BRCA2* (Fig. 6), PolyPhen-2 HumDiv predicted damaging effects in 44.99% (N = 2079) and benign effects in 55.01% (N = 2542) tested VUS. PolyPhen-2 HumVar predicted damaging effects in 31.16% (N = 1440) and benign effects in 68.84% (N = 3181) tested VUS. SIFT predicted damaging effects in 51.48% (N = 2379) and tolerated effects in 48.52% (N = 2242) of tested VUS. Agreement was achieved in prediction outcomes from the three tested approaches in 68.97% (N = 3187) of the tested VUS with 1872 and 1315 VUS being predicted as benign and damaging, respectively (Fig. 7). On the other hand, PolyPhen-2 HumDiv achieved highest consensus with PolyPhen-2 HumVar in predicting benign VUS. However, for the damaging predictions, highest consensus was achieved between SIFT and PolyPhen-2 HumDiv (Fig. 8). Notably, about 15.70% (N = 519) and 33.28% (N = 915) of predictions observed were unique (outstanding predictions given by only one among three algorithm) for benign and damaging categories, respectively. Similar to the observations on *BRCA1* VUS, higher concordance (consensus in at least two out of three tested approaches) was achieved for benign prediction in *BRCA2* VUS.

## Discussions

The current study compared the prediction performance of PolyPhen-2 HumDiv, PolyPhen-2 HumVar and SIFT using benchmark datasets for *BRCA1* and *BRCA2* genes. The study was further extended to evaluate their practical use to predict the pathogenicity of reported VUS in the context of the *BRCA1* and *BRCA2* genes. Analysis was based on the curated variants with expert review from the ClinVar^18^ which is widely referred by genetic laboratory personnel, clinicians and scientists nowadays for interpreting the molecular genetic testing results.

Results from this study show differences between the performance of PolyPhen-2 HumDiv, PolyPhen-2 HumVar and SIFT. Of the missense variants with known clinical significance (classified benign or pathogenic), there was concordant agreement achieved in the predictions in 96 (58.18%) of 165 *BRCA1* and in 95 (66.90%) of 142 *BRCA2* variants for all three approaches used, respectively. The discordant predictions are attributed to the inherent characteristics of different systems used by different algorithms. The PolyPhen-2 combines information from protein sequence and structure to analyse missense variants. However, two different datasets had been used to train the HumDiv and HumVar, hence resulting in different analytical approaches to a given missense variant^20^. HumDiv data is mainly used to discriminate the rare alleles in complex disease phenotypes and in natural selection. On the other hand, HumVar data is more relevant in analysing variants of Mendelian disorders, which more stringently recognise highly disease-causing mutations from benign polymorphism existing in the normal population. The hereditary breast cancers do not clearly fit into either category of complex or Mendelian disorder because of the incomplete penetrance imposed by the two pre-disposing genes. The HumVar had shown higher overall accuracy than HumDiv i.e. 66.67% versus 60.00% and 77.46% versus 61.27% for *BRCA1* and *BRCA2* missense variants, respectively. Notably, both HumDiv and HumVar had higher NPV (> 85%) than PPV (< 35%) indicating the PolyPhen-2 had given more confident results in predicting benign variants. When SIFT was evaluated along with PolyPhen-2 HumDiv and HumVar, it had shown top performance in terms of NPV (100%) and sensitivity (100%) for *BRCA1* and *BRCA2*. Notably, HumDiv had also shown 100% NPV and 100% sensitivity for *BRCA2*. Hence, in this study although some similar performances were observed, these algorithms were noted to have varied predictive performances for *BRCA1* and *BRCA2* missense variants, respectively.

Notably, the NPV of the three tested approaches were all above 85% (Table 1). However, the other performances including PPV, specificity and accuracy were generally low (< 80%), hence, rendering clinical applicability of these approaches unsuitable. A limitation of the current study is the small size of datasets of tested missense variants with known clinical significance for *BRCA1* (Benign, N = 131; Pathogenic, N = 34) and *BRCA2* (Benign, N = 129; Pathogenic, N = 13), respectively.

Genetic testing for *BRCA1* and *BRCA2* genes can be important to provide actionable results to the clinicians and genetic counsellors who are directly dealing with the patients and their relatives. The utility of such testing is strongly dependent on the interpretation of variants identified. The current laboratory practice in variant interpretation include searching the published literature and online curated databases for evidence of pathogenicity. Frequently the identified missense variants are novel and are not catalogued in the existing publicly available databases. In vitro assays are performed by research laboratories to study the functional impact of the missense variant to the protein, however, this is beyond the ability of most clinical laboratories. The functional tests for *BRCA1* and *BRCA2* are generally time-consuming, laborious and costly. Studies of familial co-segregation of variant with cancer is feasible by providing carrier testing but such extended testing involves genotyping of many relatives collectively before conclusion can be firmly drawn. Again, such strategy is more applicable in the research setting.

Although lacking evidence from functional analysis and familial co-segregation with cancer, a rare missense variant is not always considered as VUS. Apart from very frequent polymorphic variants, according to ENIGMA *BRCA1/2* gene variant classification criteria, a missense variant could be classified as class 1^13^ if it has a prior probability of pathogenicity ≤ 0.02 from clinically calibrated bioinformatic analyses and an allele frequency ≥ 0.001 and < 0.01 in large outbred control reference groups. Reporting VUS in the clinical report could lead to problematic interpretation since such results cannot be readily utilised to identify the at-risk family members and provide indication for increased surveillances. Such uninformative results could also possibly raise the misperception in the patients and family members that they are at higher risk of developing cancers.

In the current study, low consensus was noted when the three approaches were used to predict the pathogenicity of the VUS. Agreement was achieved in prediction outcomes from the three tested approaches in 55.04% and 68.97% of the VUS for *BRCA1* and *BRCA2*, respectively. The lack of concordance in prediction outcomes can cause confusion in interpreting the significance of the missense variants. Previous comparative studies which evaluated other in silico algorithms suggested that combining several prediction tools could improve the prediction performance^22^, however, the study by Walters-Sen et al.^23^ had reported the opposite.

There had been several other studies conducted in silico analysis on *BRCA1* and *BRCA2* missense variants. Based on the datasets from a commercial testing laboratory, Kerr et al.^24^ reported that SIFT presented 100% sensitivity and NPV in predicting both *BRCA1* and *BRCA2* variants, a similar finding as ours. Ernst et al.^25^ also showed 100% sensitivity for SIFT predictions on both *BRCA1* and *BRCA2* variants, which was also in congruent with the findings from our study. However, for accuracy we have shown that PolyPhen-2 had better performance than SIFT as compared to the studies by Kerr et al. and Ernst et al. Another multifactorial likelihood study by Parsons et al. had reported quantitative analysis of *BRCA1* and *BRCA2* variants in which bioinformatic prediction of variant effect was part of the analysis^26^. Parsons et al. showed that the incorporation of clinical information can improve variant curation over purely bioinformatic approaches. This again highlights that bioinformatic approaches can at most serve as adjunct evidence in variant classification, and should not be solely considered in calling variant pathogenicity.

In conclusion, the performances of PolyPhen-2 and SIFT in predicting functional impacts varied across a clinical dataset of *BRCA1* and *BRCA2* missense variants. Lack of high concordance in prediction outcomes highlighted their limited clinical application in classifying the pathogenicity of VUS identified through molecular testing of *BRCA1* and *BRCA2*.

## Methodology

### Mining of *BRCA1* and *BRCA2* missense variants

ClinVar is a public depository of genetic variants allowing submissions of curations with clinical significance. ClinVar is a freely accessible database (https://www.ncbi.nlm.nih.gov/clinvar/). The variants of the *BRCA1* and *BRCA2* genes as of March 16, 2021 were downloaded after applying filters including “missense” for “molecular consequence” and “expert panel” for “review status”. The datasets were imported into a spreadsheet software, Microsoft Excel for Mac, visualized and analysed. Entries of different DNA alterations representing the identical missense protein change were removed. For *BRCA1*, a missense variant, c.5074G > C (p.Asp1692His) was excluded from analysis due to its proven effects on mRNA splicing instead of amino acid substitution^27,28^. A total of 307 expert reviewed entries consisting of 34 pathogenic and 131 benign *BRCA1*, and 13 pathogenic and 129 benign *BRCA2* variants were subject to in silico analysis for functional predictions.

The missense variants of unknown significance (VUS) were also sought for the *BRCA1* and *BRCA2* genes in ClinVar by applying filters including “missense” for “molecular consequence” and “uncertain significance” for “clinical significance”. Duplicate entries with identical missense protein change were removed. There were 2440 *BRCA1* and 4621 *BRCA2* VUS included in this study.

### In silico analysis using PolyPhen-2

PolyPhen-2 is an online tool for prediction of the functional consequences of an amino acid substitution on a human protein. Polyphen-2 web interface was accessed at http://genetics.bwh.harvard.edu/pph2/index.shtml. Batch query mode was used for analysis. The query line of missense variant was outlined according to the format:

“# Protein ID PositionAA1 AA1”. The protein ID of *BRCA1* and *BRCA2* are NP_009225 and NP_000050, respectively. Both HumDiv- and HumVar-trained PolyPhen-2 models were used in this study. Each analysed variants is classified as benign (score ≤ 0.5), possibly damaging (0.5 < score ≤ 0.9), or probably damaging (score > 0.9) according to the predetermined thresholds of False Positive Rate (FPR) for each of the two models (HumDiv and HumVar).

### In silico analysis using SIFT

SIFT is an online tool uses sequence homology to judge the functional impact of missense variants. SIFT web interface was accessed at http://sift.jcvi.org/. SIFT Human Protein was selected. The query line of missense was outlined by the Protein Ensembl ENSP ID followed by the specified substitution: “ENSP ID, substitution”. The Ensembl ENSP ID used for *BRCA1* and *BRCA2* were ENSP00000350283 and ENSP00000439902, respectively. The batch protein tool was used to multiple queries. SIFT give prediction outcomes for missense variants as damaging (score < 0.05) and tolerated (score ≥ 0.05).

### Evaluation of prediction results of variants with known clinical significance

The performance of the PolyPhen-2 and SIFT was analysed by comparing the statistical calculations:

$$\begin{aligned} & {\text{Positive}}\;{\text{Predictive}}\;{\text{Value}}\;\left( {{\text{PPV}}} \right) = {\text{TP}}/\left( {{\text{TP}} + {\text{FP}}} \right) \\ & {\text{Negative}}\;{\text{Predictive}}\;{\text{Value}}\;\left( {{\text{NPV}}} \right) = {\text{TN}}/\left( {{\text{TN}} + {\text{FN}}} \right) \\ & {\text{Sensitivity}} = {\text{TP}}/\left( {{\text{TP}} + {\text{FN}}} \right) \\ & {\text{Specificity}} = {\text{TN}}/\left( {{\text{FP}} + {\text{TN}}} \right) \\ & {\text{Accuracy}} = \left( {{\text{TP}} + {\text{TN}}} \right)/\left( {{\text{TP}} + {\text{FP}} + {\text{TN}} + {\text{FN}}} \right) \\ \end{aligned}$$

For these calculations, TP or True Positives are pathogenic variants with CLINVAR expert review called as possibly damaging or probably damaging by PolyPhen-2, and damaging by SIFT, respectively. FP or False Positives are benign variants called as possibly damaging or probably damaging by PolyPhen-2, and damaging by SIFT, respectively. TN or True Negatives are benign variants called as benign by PolyPhen-2, and tolerated by SIFT, respectively. FN or False Negatives are pathogenic variants called as benign by PolyPhen-2, and tolerated by SIFT, respectively. Calculations were performed using MedCal diagnostic test evaluator calculator.

## Supplementary Information

Supplementary Information 1.

Supplementary Information 2.
