# Supplementary material for: In silico analysis of BRCA1 and BRCA2 missense variants and the relevance in molecular genetic testing
Source: Sci Rep. 2021 May 27;11:11114. doi: 10.1038/s41598-021-88586-w (PMC8160182; doi:10.1038/s41598-021-88586-w)
Supplement: Supplementary file 1 — Supplementary Information 1. [file 41598_2021_88586_MOESM1_ESM.docx]

**Supplementary Table 1** Prediction results from PolyPhen-2 (HumDiV), PolyPhen-2 (HumVar) and SIFT on *BRCA1* missense variants with known clinical significance.

| Nucleotide change (NM_007294.4) | Protein change (NP_009225.1) | ClinVar classification | PolyPhen-2 HumDiv | | PolyPhen-2 HumVar | | SIFT | |
| --- | --- | --- | --- | --- | --- | --- | --- | --- |
|  |  |  | Classification | Score | Classification | Score | Classification | Score |
| c.53T>C | p.Met18Thr | Pathogenic | Possibly damaging | 0.801 | Benign | 0.344 | Damaging | 0 |
| c.65T>C | p.Leu22Ser | Pathogenic | Probably damaging | 0.979 | Possibly damaging | 0.702 | Damaging | 0 |
| c.110C>A | p.Thr37Lys | Pathogenic | Probably damaging | 0.979 | Possibly damaging | 0.777 | Damaging | 0 |
| c.115T>C | p.Cys39Arg | Pathogenic | Probably damaging | 0.987 | Possibly damaging | 0.879 | Damaging | 0 |
| c.122A>G | p.His41Arg | Pathogenic | Probably damaging | 0.959 | Possibly damaging | 0.542 | Damaging | 0 |
| c.130T>A | p.Cys44Ser | Pathogenic | Probably damaging | 0.979 | Possibly damaging | 0.76 | Damaging | 0 |
| c.131G>A | p.Cys44Tyr | Pathogenic | Probably damaging | 0.994 | Possibly damaging | 0.84 | Damaging | 0 |
| c.131G>T | p.Cys44Phe | Pathogenic | Probably damaging | 0.994 | Possibly damaging | 0.879 | Damaging | 0 |
| c.140G>A | p.Cys47Tyr | Pathogenic | Possibly damaging | 0.677 | Benign | 0.223 | Damaging | 0 |
| c.181T>G | p.Cys61Gly | Pathogenic | Benign | 0.116 | Benign | 0.026 | Damaging | 0 |
| c.191G>A | p.Cys64Tyr | Pathogenic | Probably damaging | 0.999 | Probably damaging | 0.972 | Damaging | 0 |
| c.211A>G | p.Arg71Gly | Pathogenic | Possibly damaging | 0.956 | Benign | 0.444 | Damaging | 0 |
| c.4484G>C | p.Arg1495Thr | Pathogenic | Benign | 0.094 | Benign | 0.026 | Damaging | 0.01 |
| c.4675G>A | p.Glu1559Lys | Pathogenic | Benign | 0.011 | Benign | 0.006 | Damaging | 0 |
| c.5053A>G | p.Thr1685Ala | Pathogenic | Possibly damaging | 0.53 | Benign | 0.183 | Damaging | 0 |
| c.5054C>T | p.Thr1685Ile | Pathogenic | Possibly damaging | 0.89 | Benign | 0.329 | Damaging | 0 |
| c.5089T>C | p.Cys1697Arg | Pathogenic | Possibly damaging | 0.915 | Possibly damaging | 0.502 | Damaging | 0 |
| c.5095C>T | p.Arg1699Trp | Pathogenic | Probably damaging | 1 | Probably damaging | 0.992 | Damaging | 0 |
| c.5096G>A | p.Arg1699Gln | Pathogenic | Probably damaging | 1 | Probably damaging | 0.915 | Damaging | 0 |
| c.5117G>A | p.Gly1706Glu | Pathogenic | Probably damaging | 0.998 | Possibly damaging | 0.904 | Damaging | 0 |
| c.5123C>A | p.Ala1708Glu | Pathogenic | Probably damaging | 1 | Probably damaging | 0.972 | Damaging | 0 |
| c.5143A>C | p.Ser1715Arg | Pathogenic | Possibly damaging | 0.786 | Benign | 0.33 | Damaging | 0 |
| c.5144G>A | p.Ser1715Asn | Pathogenic | Probably damaging | 0.976 | Possibly damaging | 0.552 | Damaging | 0 |
| c.5207T>C | p.Val1736Ala | Pathogenic | Possibly damaging | 0.802 | Benign | 0.38 | Damaging | 0 |
| c.5212G>A | p.Gly1738Arg | Pathogenic | Probably damaging | 0.999 | Probably damaging | 0.942 | Damaging | 0 |
| c.5291T>C | p.Leu1764Pro | Pathogenic | Probably damaging | 0.999 | Probably damaging | 0.937 | Damaging | 0 |
| c.5297T>G | p.Ile1766Ser | Pathogenic | Possibly damaging | 0.882 | Possibly damaging | 0.844 | Damaging | 0 |
| c.5309G>T | p.Gly1770Val | Pathogenic | Possibly damaging | 0.583 | Benign | 0.055 | Damaging | 0 |
| c.5324T>A | p.Met1775Lys | Pathogenic | Probably damaging | 0.99 | Possibly damaging | 0.798 | Damaging | 0 |
| c.5324T>G | p.Met1775Arg | Pathogenic | Probably damaging | 0.998 | Possibly damaging | 0.844 | Damaging | 0 |
| c.5363G>T | p.Gly1788Val | Pathogenic | Benign | 0.296 | Benign | 0.028 | Damaging | 0 |
| c.5509T>C | p.Trp1837Arg | Pathogenic | Probably damaging | 1 | Probably damaging | 1 | Damaging | 0 |
| c.5513T>A | p.Val1838Glu | Pathogenic | Probably damaging | 1 | Probably damaging | 1 | Damaging | 0 |
| c.5516T>C | p.Leu1839Ser | Pathogenic | Probably damaging | 1 | Probably damaging | 1 | Damaging | 0 |
| c.133A>C | p.Lys45Gln | Benign | benign | 0.317 | Benign | 0.17 | Damaging | 0 |
| c.199G>T | p.Asp67Tyr | Benign | Possibly damaging | 0.868 | Benign | 0.24 | Damaging | 0 |
| c.305C>G | p.Ala102Gly | Benign | Benign | 0.057 | Benign | 0.023 | Damaging | 0.01 |
| c.314A>G | p.Tyr105Cys | Benign | Benign | 0.434 | Benign | 0.196 | Damaging | 0 |
| c.370A>G | p.Ile124Val | Benign | Possibly damaging | 0.9 | Benign | 0.344 | Tolerated | 0.27 |
| c.396C>A | p.Asn132Lys | Benign | Possibly damaging | 0.844 | Benign | 0.23 | Damaging | 0 |
| c.397C>T | p.Arg133Cys | Benign | Probably damaging | 1 | Probably damaging | 0.925 | Damaging | 0 |
| c.425C>A | p.Pro142His | Benign | Probably damaging | 0.996 | Possibly damaging | 0.85 | Damaging | 0 |
| c.427G>A | p.Glu143Lys | Benign | Benign | 0.019 | Benign | 0.006 | Tolerated | 0.12 |
| c.463C>G | p.Gln155Glu | Benign | Benign | 0 | Benign | 0 | Tolerated | 0.3 |
| c.508C>T | p.Arg170Trp | Benign | Benign | 0 | Benign | 0 | Damaging | 0.05 |
| c.536A>G | p.Tyr179Cys | Benign | Probably damaging | 0.999 | Possibly damaging | 0.846 | Damaging | 0 |
| c.557C>A | p.Ser186Tyr | Benign | Probably damaging | 1 | Probably damaging | 0.956 | Damaging | 0 |
| c.571G>A | p.Val191Ile | Benign | Benign | 0.004 | Benign | 0.001 | Tolerated | 0.1 |
| c.641A>G | p.Asp214Gly | Benign | Benign | 0.001 | Benign | 0.001 | Tolerated | 0.62 |
| c.716A>G | p.His239Arg | Benign | Benign | 0.246 | Benign | 0.088 | Tolerated | 0.1 |
| c.736T>G | p.Leu246Val | Benign | Probably damaging | 1 | Probably damaging | 0.981 | Tolerated | 0.38 |
| c.823G>A | p.Gly275Ser | Benign | Probably damaging | 0.995 | Probably damaging | 0.927 | Tolerated | 0.08 |
| c.824G>A | p.Gly275Asp | Benign | Probably damaging | 1 | Probably damaging | 0.994 | Tolerated | 0.22 |
| c.827C>G | p.Thr276Arg | Benign | Probably damaging | 0.978 | Possibly damaging | 0.844 | Tolerated | 0.09 |
| c.839C>G | p.Ala280Gly | Benign | Probably damaging | 0.989 | Possibly damaging | 0.831 | Damaging | 0.04 |
| c.891G>A | p.Met297Ile | Benign | Benign | 0.074 | Benign | 0.06 | Tolerated | 0.13 |
| c.946A>G | p.Ser316Gly | Benign | Probably damaging | 1 | Probably damaging | 0.991 | Damaging | 0 |
| c.997A>G | p.Thr333Ala | Benign | Benign | 0.009 | Benign | 0.019 | Tolerated | 0.14 |
| c.1001C>T | p.Pro334Leu | Benign | Possibly damaging | 0.584 | Benign | 0.263 | Tolerated | 0.06 |
| c.1036C>T | p.Pro346Ser | Benign | Benign | 0.017 | Benign | 0.048 | Tolerated | 0.83 |
| c.1067A>G | p.Gln356Arg | Benign | Probably damaging | 0.998 | Probably damaging | 0.988 | Damaging | 0.01 |
| c.1105G>A | p.Asp369Asn | Benign | Probably damaging | 0.966 | Possibly damaging | 0.838 | Damaging | 0.04 |
| c.1159T>A | p.Ser387Thr | Benign | Probably damaging | 0.974 | Possibly damaging | 0.855 | Damaging | 0.02 |
| c.1383T>A | p.Phe461Leu | Benign | Probably damaging | 1 | Probably damaging | 1 | Damaging | 0 |
| c.1418A>G | p.Asn473Ser | Benign | Possibly damaging | 0.736 | Possibly damaging | 0.641 | Tolerated | 0.15 |
| c.1418A>T | p.Asn473Ile | Benign | Probably damaging | 0.997 | Probably damaging | 0.971 | Damaging | 0 |
| c.1456T>C | p.Phe486Leu | Benign | Benign | 0 | Benign | 0.008 | Tolerated | 0.61 |
| c.1486C>T | p.Arg496Cys | Benign | Benign | 0.004 | Benign | 0.009 | Tolerated | 0.24 |
| c.1487G>A | p.Arg496His | Benign | Benign | 0.001 | Benign | 0.004 | Tolerated | 0.92 |
| c.1511G>A | p.Arg504His | Benign | Possibly damaging | 0.692 | Benign | 0.344 | Tolerated | 0.08 |
| c.1534C>T | p.Leu512Phe | Benign | Probably damaging | 1 | Probably damaging | 0.998 | Damaging | 0 |
| c.1616C>T | p.Thr539Met | Benign | Benign | 0 | Benign | 0.001 | Tolerated | 1 |
| c.1648A>C | p.Asn550His | Benign | Probably damaging | 0.996 | Possibly damaging | 0.88 | Damaging | 0.01 |
| c.1703C>T | p.Pro568Leu | Benign | Benign | 0.031 | Benign | 0.041 | Damaging | 0.01 |
| c.1789G>A | p.Glu597Lys | Benign | Probably damaging | 1 | Probably damaging | 0.992 | Tolerated | 0.11 |
| c.1834A>G | p.Arg612Gly | Benign | Possibly damaging | 0.686 | Benign | 0.323 | Damaging | 0 |
| c.1865C>T | p.Ala622Val | Benign | Possibly damaging | 0.63 | Benign | 0.15 | Damaging | 0.05 |
| c.1924G>C | p.Asp642His | Benign | Probably damaging | 0.997 | Probably damaging | 0.945 | Damaging | 0.01 |
| c.1927A>G | p.Ser643Gly | Benign | Possibly damaging | 0.927 | Possibly damaging | 0.677 | Damaging | 0.02 |
| c.2002C>T | p.Leu668Phe | Benign | Possibly damaging | 0.812 | Possibly damaging | 0.679 | Damaging | 0.05 |
| c.2077G>A | p.Asp693Asn | Benign | Benign | 0 | Benign | 0.01 | Tolerated | 0.16 |
| c.2083G>T | p.Asp695Tyr | Benign | Probably damaging | 0.976 | Possibly damaging | 0.836 | Damaging | 0.01 |
| c.2167A>G | p.Asn723Asp | Benign | Benign | 0.164 | Benign | 0.119 | Tolerated | 0.15 |
| c.2180C>T | p.Pro727Leu | Benign | Benign | 0.001 | Benign | 0.016 | Tolerated | 0.37 |
| c.2286A>T | p.Arg762Ser | Benign | Benign | 0.19 | Possibly damaging | 0.588 | Tolerated | 0.09 |
| c.2315T>C | p.Val772Ala | Benign | Possibly damaging | 0.848 | Probably damaging | 0.928 | Damaging | 0.01 |
| c.2351C>T | p.Ser784Leu | Benign | Probably damaging | 0.995 | Probably damaging | 0.954 | Damaging | 0.02 |
| c.2412G>C | p.Gln804His | Benign | Benign | 0.047 | Benign | 0.285 | Tolerated | 0.16 |
| c.2428A>T | p.Asn810Tyr | Benign | Probably damaging | 1 | Probably damaging | 0.992 | Damaging | 0 |
| c.2458A>G | p.Lys820Glu | Benign | Benign | 0.006 | Benign | 0.108 | Tolerated | 0.29 |
| c.2477C>A | p.Thr826Lys | Benign | Possibly damaging | 0.954 | Probably damaging | 0.968 | Damaging | 0.05 |
| c.2521C>T | p.Arg841Trp | Benign | Benign | 0.001 | Benign | 0.006 | Damaging | 0 |
| c.2525A>G | p.Glu842Gly | Benign | Possibly damaging | 0.93 | Possibly damaging | 0.819 | Damaging | 0.02 |
| c.2566T>C | p.Tyr856His | Benign | Possibly damaging | 0.73 | Possibly damaging | 0.733 | Damaging | 0.02 |
| c.2584A>G | p.Lys862Glu | Benign | Probably damaging | 0.974 | Possibly damaging | 0.876 | Damaging | 0 |
| c.2596C>T | p.Arg866Cys | Benign | Probably damaging | 1 | Probably damaging | 1 | Damaging | 0 |
| c.2597G>A | p.Arg866His | Benign | Probably damaging | 1 | Probably damaging | 0.986 | Damaging | 0.01 |
| c.2612C>T | p.Pro871Leu | Benign | Benign | 0 | Benign | 0 | Tolerated | 1 |
| c.2669G>T | p.Gly890Val | Benign | Benign | 0.002 | Benign | 0.007 | Tolerated | 0.31 |
| c.2758G>A | p.Val920Ile | Benign | Possibly damaging | 0.549 | Benign | 0.34 | Tolerated | 0.07 |
| c.2773A>C | p.Ile925Leu | Benign | Benign | 0.01 | Benign | 0.038 | Damaging | 0.02 |
| c.2798G>C | p.Gly933Ala | Benign | Benign | 0.001 | Benign | 0.016 | Tolerated | 0.11 |
| c.2884G>A | p.Glu962Lys | Benign | Possibly damaging | 0.731 | Possibly damaging | 0.474 | Tolerated | 0.15 |
| c.2912A>G | p.His971Arg | Benign | Benign | 0.036 | Benign | 0.098 | Tolerated | 0.25 |
| c.3022A>G | p.Met1008Val | Benign | Benign | 0 | Benign | 0 | Tolerated | 0.92 |
| c.3024G>A | p.Met1008Ile | Benign | Benign | 0 | Benign | 0.001 | Tolerated | 0.56 |
| c.3082C>T | p.Arg1028Cys | Benign | Possibly damaging | 0.587 | Benign | 0.276 | Tolerated | 0.07 |
| c.3083G>A | p.Arg1028His | Benign | Benign | 0 | Benign | 0 | Tolerated | 0.22 |
| c.3113A>G | p.Glu1038Gly | Benign | Possibly damaging | 0.936 | Possibly damaging | 0.606 | Damaging | 0.04 |
| c.3119G>A | p.Ser1040Asn | Benign | Probably damaging | 0.974 | Possibly damaging | 0.831 | Tolerated | 0.06 |
| c.3130A>G | p.Ile1044Val | Benign | Benign | 0.016 | Benign | 0.009 | Tolerated | 0.39 |
| c.3143G>T | p.Gly1048Val | Benign | Possibly damaging | 0.861 | Possibly damaging | 0.677 | Tolerated | 0.76 |
| c.3296C>T | p.Pro1099Leu | Benign | Probably damaging | 0.989 | Possibly damaging | 0.677 | Damaging | 0.02 |
| c.3302G>A | p.Ser1101Asn | Benign | Possibly damaging | 0.765 | Benign | 0.263 | Tolerated | 0.28 |
| c.3327A>C | p.Lys1109Asn | Benign | Probably damaging | 0.989 | Possibly damaging | 0.665 | Damaging | 0 |
| c.3416G>T | p.Ser1139Ile | Benign | Benign | 0.386 | Benign | 0.21 | Damaging | 0.01 |
| c.3418A>G | p.Ser1140Gly | Benign | Benign | 0.002 | Benign | 0.008 | Tolerated | 0.49 |
| c.3448C>T | p.Pro1150Ser | Benign | Probably damaging | 0.995 | Probably damaging | 0.968 | Damaging | 0.01 |
| c.3463G>C | p.Asp1155His | Benign | Possibly damaging | 0.604 | Benign | 0.245 | Damaging | 0.05 |
| c.3541G>A | p.Val1181Ile | Benign | Benign | 0.007 | Benign | 0.013 | Tolerated | 0.27 |
| c.3548A>G | p.Lys1183Arg | Benign | Benign | 0 | Benign | 0.001 | Tolerated | 0.94 |
| c.3600G>T | p.Gln1200His | Benign | Benign | 0.207 | Benign | 0.179 | Damaging | 0.04 |
| c.3608G>A | p.Arg1203Gln | Benign | Benign | 0 | Benign | 0 | Tolerated | 0.85 |
| c.3640G>A | p.Glu1214Lys | Benign | Possibly damaging | 0.651 | Benign | 0.165 | Damaging | 0 |
| c.3657G>C | p.Glu1219Asp | Benign | Benign | 0.009 | Benign | 0.011 | Tolerated | 0.12 |
| c.3708T>G | p.Asn1236Lys | Benign | Benign | 0.031 | Benign | 0.006 | Damaging | 0.01 |
| c.3713C>T | p.Pro1238Leu | Benign | Benign | 0.264 | Benign | 0.03 | Damaging | 0.05 |
| c.3724A>G | p.Thr1242Ala | Benign | Benign | 0.146 | Benign | 0.057 | Tolerated | 0.09 |
| c.3739G>A | p.Val1247Ile | Benign | Benign | 0.154 | Benign | 0.01 | Tolerated | 0.47 |
| c.3748G>A | p.Glu1250Lys | Benign | Possibly damaging | 0.84 | Benign | 0.089 | Tolerated | 0.07 |
| c.3797G>C | p.Ser1266Thr | Benign | Benign | 0.144 | Benign | 0.014 | Damaging | 0.02 |
| c.3823A>G | p.Ile1275Val | Benign | Benign | 0.019 | Benign | 0.01 | Tolerated | 0.22 |
| c.4039A>G | p.Arg1347Gly | Benign | Benign | 0.255 | Benign | 0.071 | Damaging | 0.05 |
| c.4046C>T | p.Thr1349Met | Benign | Possibly damaging | 0.575 | Benign | 0.107 | Damaging | 0.04 |
| c.4081A>T | p.Met1361Leu | Benign | Benign | 0 | Benign | 0.001 | Tolerated | 0.31 |
| c.4132G>A | p.Val1378Ile | Benign | Benign | 0.001 | Benign | 0 | Tolerated | 0.2 |
| c.4204C>T | p.His1402Tyr | Benign | Benign | 0.019 | Benign | 0.023 | Tolerated | 0.1 |
| c.4255G>C | p.Glu1419Gln | Benign | Benign | 0.031 | Benign | 0.016 | Damaging | 0 |
| c.4327C>G | p.Arg1443Gly | Benign | Benign | 0.03 | Benign | 0.018 | Tolerated | 0.11 |
| c.4402A>C | p.Asn1468His | Benign | Possibly damaging | 0.641 | Benign | 0.275 | Damaging | 0 |
| c.4520G>C | p.Arg1507Thr | Benign | Benign | 0.094 | Benign | 0.049 | Damaging | 0.01 |
| c.4535G>T | p.Ser1512Ile | Benign | Benign | 0.337 | Benign | 0.133 | Damaging | 0.01 |
| c.4600G>A | p.Val1534Met | Benign | Benign | 0.07 | Benign | 0.006 | Tolerated | 0.36 |
| c.4636G>A | p.Asp1546Asn | Benign | Possibly damaging | 0.824 | Benign | 0.056 | Tolerated | 0.26 |
| c.4636G>T | p.Asp1546Tyr | Benign | Probably damaging | 0.979 | Possibly damaging | 0.607 | Damaging | 0 |
| c.4691T>C | p.Leu1564Pro | Benign | Possibly damaging | 0.828 | Benign | 0.221 | Tolerated | 0.21 |
| c.4816A>G | p.Lys1606Glu | Benign | Benign | 0 | Benign | 0 | Damaging | 0 |
| c.4837A>G | p.Ser1613Gly | Benign | Benign | 0.255 | Benign | 0.038 | Damaging | 0 |
| c.4840C>T | p.Pro1614Ser | Benign | Benign | 0.001 | Benign | 0.001 | Damaging | 0.03 |
| c.4883T>C | p.Met1628Thr | Benign | Benign | 0.039 | Benign | 0.028 | Tolerated | 0.16 |
| c.4910C>T | p.Pro1637Leu | Benign | Benign | 0.364 | Benign | 0.057 | Damaging | 0 |
| c.4955T>C | p.Met1652Thr | Benign | Benign | 0.004 | Benign | 0.053 | Damaging | 0 |
| c.4956G>A | p.Met1652Ile | Benign | Benign | 0.001 | Benign | 0.007 | Tolerated | 0.12 |
| c.4985T>C | p.Phe1662Ser | Benign | Benign | 0.327 | Benign | 0.072 | Damaging | 0.02 |
| c.4991T>C | p.Leu1664Pro | Benign | Benign | 0.005 | Benign | 0.003 | Tolerated | 0.07 |
| c.5024C>T | p.Thr1675Ile | Benign | Benign | 0.011 | Benign | 0.019 | Damaging | 0 |
| c.5044G>A | p.Glu1682Lys | Benign | Benign | 0.102 | Benign | 0.024 | Damaging | 0 |
| c.5117G>C | p.Gly1706Ala | Benign | Probably damaging | 0.965 | Possibly damaging | 0.577 | Damaging | 0 |
| c.5158A>G | p.Thr1720Ala | Benign | Benign | 0.021 | Benign | 0.066 | Damaging | 0.01 |
| c.5198A>G | p.Asp1733Gly | Benign | Possibly damaging | 0.734 | Benign | 0.197 | Damaging | 0 |
| c.5252G>A | p.Arg1751Gln | Benign | Possibly damaging | 0.946 | Benign | 0.032 | Damaging | 0 |
| c.5411T>A | p.Val1804Asp | Benign | Benign | 0.001 | Benign | 0.001 | Damaging | 0.02 |
| c.5531T>G | p.Leu1844Arg | Benign | Probably damaging | 0.999 | Probably damaging | 0.949 | Damaging | 0 |
| c.5572A>C | p.Ile1858Leu | Benign | Benign | 0 | Benign | 0.001 | Damaging | 0.02 |
| c.5576C>G | p.Pro1859Arg | Benign | Possibly damaging | 0.764 | Benign | 0.272 | Damaging | 0 |
