# Supplementary material for: In silico analysis of BRCA1 and BRCA2 missense variants and the relevance in molecular genetic testing
Source: Sci Rep. 2021 May 27;11:11114. doi: 10.1038/s41598-021-88586-w (PMC8160182; doi:10.1038/s41598-021-88586-w)
Supplement: Supplementary file 2 — Supplementary Information 2. [file 41598_2021_88586_MOESM2_ESM.docx]

**Supplementary Table 2** Prediction results from PolyPhen-2 (HumDiV), PolyPhen-2 (HumVar) and SIFT on *BRCA2* missense variants with known clinical significance.

| Nucleotide change (NM_007294.4) | Protein change (NP_009225.1) | ClinVar classification | PolyPhen-2 HumDiv | | PolyPhen-2 HumVar | | SIFT | |
| --- | --- | --- | --- | --- | --- | --- | --- | --- |
|  |  |  | Classification | Score | Classification | Score | Classification | Score |
| c.7007G>A | p.Arg2336His | Pathogenic | Possibly damaging | 0.733 | Benign | 0.083 | Damaging | 0.05 |
| c.7878G>C | p.Trp2626Cys | Pathogenic | Probably damaging | 1 | Probably damaging | 1 | Damaging | 0 |
| c.7879A>T | p.Ile2627Phe | Pathogenic | Probably damaging | 1 | Probably damaging | 0.996 | Damaging | 0 |
| c.7975A>G | p.Arg2659Gly | Pathogenic | Probably damaging | 1 | Probably damaging | 1 | Damaging | 0.01 |
| c.7976G>C | p.Arg2659Thr | Pathogenic | Probably damaging | 1 | Probably damaging | 1 | Damaging | 0.01 |
| c.7988A>T | p.Glu2663Val | Pathogenic | Probably damaging | 1 | Probably damaging | 1 | Damaging | 0.01 |
| c.8023A>G | p.Ile2675Val | Pathogenic | Probably damaging | 0.999 | Probably damaging | 0.995 | Damaging | 0 |
| c.8165C>G | p.Thr2722Arg | Pathogenic | Probably damaging | 1 | Probably damaging | 1 | Damaging | 0 |
| c.8167G>C | p.Asp2723His | Pathogenic | Probably damaging | 1 | Probably damaging | 1 | Damaging | 0 |
| c.8168A>G | p.Asp2723Gly | Pathogenic | Probably damaging | 1 | Probably damaging | 1 | Damaging | 0 |
| c.8243G>A | p.Gly2748Asp | Pathogenic | Probably damaging | 1 | Probably damaging | 1 | Damaging | 0 |
| c.9154C>T | p.Arg3052Trp | Pathogenic | Probably damaging | 1 | Probably damaging | 1 | Damaging | 0 |
| c.9371A>T | p.Asn3124Ile | Pathogenic | Probably damaging | 1 | Probably damaging | 1 | Damaging | 0 |
| c.53G>A | p.Arg18His | Benign | Probably damaging | 0.962 | Benign | 0.222 | Tolerated | 0.256 |
| c.125A>G | p.Tyr42Cys | Benign | Benign | 0.09 | Benign | 0.028 | Tolerated | 0.113 |
| c.167A>C | p.Asn56Thr | Benign | Benign | 0.001 | Benign | 0 | Tolerated | 1 |
| c.223G>C | p.Ala75Pro | Benign | Probably damaging | 1 | Probably damaging | 0.988 | Damaging | 0.006 |
| c.322A>C | p.Asn108His | Benign | Benign | 0 | Benign | 0.001 | Tolerated | 0.11 |
| c.440A>G | p.Gln147Arg | Benign | Benign | 0 | Benign | 0.001 | Tolerated | 1 |
| c.502C>A | p.Pro168Thr | Benign | Probably damaging | 1 | Probably damaging | 0.998 | Damaging | 0 |
| c.865A>C | p.Asn289His | Benign | Benign | 0.278 | Benign | 0.034 | Damaging | 0.002 |
| c.978C>A | p.Ser326Arg | Benign | Benign | 0.001 | Benign | 0.001 | Damaging | 0.048 |
| c.1040A>G | p.Gln347Arg | Benign | Possibly damaging | 0.483 | Benign | 0.163 | Tolerated | 0.149 |
| c.1114A>C | p.Asn372His | Benign | Benign | 0 | Benign | 0 | Tolerated | 0.139 |
| c.1123C>T | p.Pro375Ser | Benign | Benign | 0.098 | Benign | 0.016 | Tolerated | 0.155 |
| c.1151C>T | p.Ser384Phe | Benign | Probably damaging | 0.994 | Possibly damaging | 0.841 | Damaging | 0.012 |
| c.1166C>A | p.Pro389Gln | Benign | Benign | 0.06 | Benign | 0.026 | Tolerated | 0.437 |
| c.1181A>C | p.Glu394Ala | Benign | Benign | 0.278 | Benign | 0.057 | Damaging | 0.027 |
| c.1354C>A | p.Leu452Ile | Benign | Benign | 0.046 | Benign | 0.014 | Tolerated | 0.135 |
| c.1385A>G | p.Glu462Gly | Benign | Probably damaging | 0.993 | Possibly damaging | 0.738 | Tolerated | 0.067 |
| c.1514T>C | p.Ile505Thr | Benign | Possibly damaging | 0.651 | Benign | 0.115 | Damaging | 0.003 |
| c.1538A>G | p.Lys513Arg | Benign | Benign | 0.072 | Benign | 0.023 | Damaging | 0.002 |
| c.1662T>G | p.Cys554Trp | Benign | Probably damaging | 0.993 | Possibly damaging | 0.72 | Damaging | 0 |
| c.1744A>C | p.Thr582Pro | Benign | Probably damaging | 0.966 | Possibly damaging | 0.641 | Damaging | 0.011 |
| c.1786G>C | p.Asp596His | Benign | Probably damaging | 0.998 | Possibly damaging | 0.87 | Damaging | 0.002 |
| c.1792A>G | p.Thr598Ala | Benign | Benign | 0.001 | Benign | 0.002 | Tolerated | 0.798 |
| c.1796C>T | p.Ser599Phe | Benign | Benign | 0.062 | Benign | 0.023 | Damaging | 0.001 |
| c.1798T>C | p.Tyr600His | Benign | Benign | 0.013 | Benign | 0.01 | Tolerated | 0.255 |
| c.1804G>A | p.Gly602Arg | Benign | Benign | 0.252 | Benign | 0.071 | Tolerated | 0.132 |
| c.1810A>G | p.Lys604Glu | Benign | Benign | 0.261 | Benign | 0.028 | Damaging | 0.007 |
| c.1865C>T | p.Ala622Val | Benign | Benign | 0.286 | Benign | 0.064 | Tolerated | 0.085 |
| c.1889C>T | p.Thr630Ile | Benign | Benign | 0.062 | Benign | 0.014 | Damaging | 0.012 |
| c.1964C>G | p.Pro655Arg | Benign | Possibly damaging | 0.561 | Benign | 0.125 | Damaging | 0.003 |
| c.2350A>G | p.Met784Val | Benign | Benign | 0 | Benign | 0 | Tolerated | 1 |
| c.2416G>C | p.Asp806His | Benign | Possibly damaging | 0.855 | Benign | 0.359 | Damaging | 0.017 |
| c.2680G>A | p.Val894Ile | Benign | Benign | 0.001 | Benign | 0.001 | Tolerated | 0.829 |
| c.2698A>G | p.Asn900Asp | Benign | Possibly damaging | 0.454 | Benign | 0.105 | Tolerated | 0.643 |
| c.2755G>A | p.Glu919Lys | Benign | Benign | 0.047 | Benign | 0.005 | Tolerated | 0.069 |
| c.2786T>C | p.Leu929Ser | Benign | Benign | 0.091 | Benign | 0.024 | Tolerated | 0.132 |
| c.2803G>C | p.Asp935His | Benign | Benign | 0.117 | Benign | 0.028 | Tolerated | 0.099 |
| c.2960A>T | p.Asn987Ile | Benign | Possibly damaging | 0.744 | Benign | 0.23 | Damaging | 0.007 |
| c.2971A>G | p.Asn991Asp | Benign | Benign | 0 | Benign | 0 | Tolerated | 1 |
| c.2987T>G | p.Leu996Arg | Benign | Benign | 0.437 | Benign | 0.186 | Tolerated | 0.14 |
| c.3055C>G | p.Leu1019Val | Benign | Benign | 0.013 | Benign | 0.017 | Tolerated | 0.141 |
| c.3304A>T | p.Asn1102Tyr | Benign | Probably damaging | 0.996 | Possibly damaging | 0.823 | Damaging | 0.01 |
| c.3515C>T | p.Ser1172Leu | Benign | Probably damaging | 0.97 | Benign | 0.334 | Damaging | 0.035 |
| c.3568C>T | p.Arg1190Trp | Benign | Possibly damaging | 0.876 | Benign | 0.153 | Damaging | 0 |
| c.3575T>G | p.Phe1192Cys | Benign | Probably damaging | 0.985 | Possibly damaging | 0.527 | Damaging | 0.015 |
| c.3581G>A | p.Gly1194Asp | Benign | Benign | 0.229 | Benign | 0.029 | Tolerated | 0.334 |
| c.3682A>G | p.Asn1228Asp | Benign | Possibly damaging | 0.896 | Possibly damaging | 0.602 | Damaging | 0.026 |
| c.3793T>A | p.Cys1265Ser | Benign | Benign | 0.004 | Benign | 0.001 | Tolerated | 0.308 |
| c.3839A>T | p.Asp1280Val | Benign | Possibly damaging | 0.728 | Benign | 0.254 | Damaging | 0.001 |
| c.3869G>A | p.Cys1290Tyr | Benign | Benign | 0.121 | Benign | 0.024 | Tolerated | 0.124 |
| c.3916G>A | p.Val1306Ile | Benign | Benign | 0.024 | Benign | 0.012 | Damaging | 0.019 |
| c.4046T>C | p.Ile1349Thr | Benign | Benign | 0.421 | Benign | 0.118 | Damaging | 0.017 |
| c.4061C>T | p.Thr1354Met | Benign | Possibly damaging | 0.876 | Benign | 0.118 | Damaging | 0.001 |
| c.4090A>C | p.Ile1364Leu | Benign | Benign | 0.001 | Benign | 0.002 | Tolerated | 0.172 |
| c.4094G>A | p.Cys1365Tyr | Benign | Benign | 0 | Benign | 0 | Tolerated | 1 |
| c.4187A>G | p.Gln1396Arg | Benign | Benign | 0.02 | Benign | 0.015 | Damaging | 0 |
| c.4241C>T | p.Thr1414Met | Benign | Benign | 0.002 | Benign | 0.001 | Tolerated | 1 |
| c.4258G>T | p.Asp1420Tyr | Benign | Benign | 0.03 | Benign | 0.011 | Damaging | 0.03 |
| c.4271C>G | p.Ser1424Cys | Benign | Benign | 0 | Benign | 0 | Tolerated | 0.219 |
| c.4570T>G | p.Phe1524Val | Benign | Probably damaging | 1 | Probably damaging | 1 | Damaging | 0.002 |
| c.4585G>A | p.Gly1529Arg | Benign | Probably damaging | 1 | Probably damaging | 1 | Damaging | 0 |
| c.4609G>A | p.Glu1537Lys | Benign | Possibly damaging | 0.835 | Benign | 0.35 | Tolerated | 0.059 |
| c.4779A>C | p.Glu1593Asp | Benign | Benign | 0.128 | Benign | 0.045 | Tolerated | 0.14 |
| c.5070A>C | p.Lys1690Asn | Benign | Probably damaging | 0.989 | Possibly damaging | 0.856 | Damaging | 0.007 |
| c.5198C>T | p.Ser1733Phe | Benign | Benign | 0.159 | Benign | 0.049 | Damaging | 0.024 |
| c.5312G>A | p.Gly1771Asp | Benign | Benign | 0.049 | Benign | 0.018 | Tolerated | 0.426 |
| c.5455C>T | p.Pro1819Ser | Benign | Benign | 0.009 | Benign | 0.007 | Tolerated | 0.322 |
| c.5552T>G | p.Ile1851Ser | Benign | Benign | 0.065 | Benign | 0.023 | Tolerated | 0.181 |
| c.5634C>G | p.Asn1878Lys | Benign | Benign | 0.015 | Benign | 0.011 | Damaging | 0.034 |
| c.5640T>G | p.Asn1880Lys | Benign | Benign | 0.167 | Benign | 0.024 | Damaging | 0.007 |
| c.5704G>A | p.Asp1902Asn | Benign | Benign | 0.055 | Benign | 0.016 | Tolerated | 0.168 |
| c.5710C>G | p.Leu1904Val | Benign | Benign | 0.001 | Benign | 0.001 | Tolerated | 0.377 |
| c.5744C>T | p.Thr1915Met | Benign | Benign | 0 | Benign | 0 | Tolerated | 0.326 |
| c.5752C>T | p.His1918Tyr | Benign | Benign | 0.001 | Benign | 0 | Tolerated | 0.616 |
| c.5753A>G | p.His1918Arg | Benign | Benign | 0.001 | Benign | 0.001 | Tolerated | 0.292 |
| c.5768A>C | p.Asp1923Ala | Benign | Benign | 0.144 | Benign | 0.035 | Damaging | 0.025 |
| c.5785A>G | p.Ile1929Val | Benign | Benign | 0.004 | Benign | 0.003 | Tolerated | 0.307 |
| c.6100C>T | p.Arg2034Cys | Benign | Possibly damaging | 0.876 | Benign | 0.118 | Damaging | 0.016 |
| c.6143A>T | p.Asn2048Ile | Benign | Probably damaging | 0.983 | Possibly damaging | 0.725 | Damaging | 0.002 |
| c.6220C>A | p.His2074Asn | Benign | Benign | 0.001 | Benign | 0.004 | Tolerated | 0.195 |
| c.6290C>T | p.Thr2097Met | Benign | Possibly damaging | 0.832 | Benign | 0.209 | Damaging | 0.024 |
| c.6322C>T | p.Arg2108Cys | Benign | Benign | 0 | Benign | 0 | Tolerated | 0.1 |
| c.6323G>A | p.Arg2108His | Benign | Benign | 0.347 | Benign | 0.042 | Tolerated | 0.147 |
| c.6338A>G | p.Asn2113Ser | Benign | Benign | 0.005 | Benign | 0.002 | Damaging | 0.028 |
| c.6347A>G | p.His2116Arg | Benign | Possibly damaging | 0.93 | Benign | 0.383 | Damaging | 0.016 |
| c.6455C>A | p.Ser2152Tyr | Benign | Possibly damaging | 0.731 | Benign | 0.311 | Damaging | 0 |
| c.6748A>G | p.Thr2250Ala | Benign | Benign | 0 | Benign | 0 | Tolerated | 0.196 |
| c.6853A>G | p.Ile2285Val | Benign | Possibly damaging | 0.612 | Benign | 0.138 | Damaging | 0.003 |
| c.6935A>T | p.Asp2312Val | Benign | Probably damaging | 0.994 | Possibly damaging | 0.865 | Damaging | 0 |
| c.6953G>A | p.Arg2318Gln | Benign | Probably damaging | 1 | Probably damaging | 0.997 | Damaging | 0 |
| c.7017G>C | p.Lys2339Asn | Benign | Benign | 0.105 | Benign | 0.042 | Damaging | 0.05 |
| c.7057G>C | p.Gly2353Arg | Benign | Probably damaging | 0.986 | Possibly damaging | 0.809 | Damaging | 0.005 |
| c.7150C>A | p.Gln2384Lys | Benign | Benign | 0 | Benign | 0 | Tolerated | 1 |
| c.7188G>T | p.Leu2396Phe | Benign | Possibly damaging | 0.855 | Benign | 0.36 | Tolerated | 0.205 |
| c.7232A>C | p.Lys2411Thr | Benign | Probably damaging | 1 | Probably damaging | 0.999 | Damaging | 0 |
| c.7307A>T | p.Asn2436Ile | Benign | Benign | 0.062 | Benign | 0.016 | Tolerated | 0.158 |
| c.7319A>G | p.His2440Arg | Benign | Benign | 0.002 | Benign | 0.003 | Tolerated | 0.305 |
| c.7397C>T | p.Ala2466Val | Benign | Possibly damaging | 0.793 | Benign | 0.355 | Tolerated | 1 |
| c.7415A>C | p.Lys2472Thr | Benign | Benign | 0.158 | Benign | 0.038 | Damaging | 0.011 |
| c.7469T>C | p.Ile2490Thr | Benign | Benign | 0.01 | Benign | 0.008 | Tolerated | 0.105 |
| c.7505G>A | p.Arg2502His | Benign | Benign | 0.004 | Benign | 0.003 | Tolerated | 0.75 |
| c.7534C>T | p.Leu2512Phe | Benign | Probably damaging | 1 | Probably damaging | 0.994 | Damaging | 0.005 |
| c.7544C>T | p.Thr2515Ile | Benign | Possibly damaging | 0.9 | Possibly damaging | 0.493 | Tolerated | 0.064 |
| c.7994A>G | p.Asp2665Gly | Benign | Probably damaging | 1 | Probably damaging | 1 | Damaging | 0 |
| c.8149G>T | p.Ala2717Ser | Benign | Possibly damaging | 0.955 | Possibly damaging | 0.763 | Tolerated | 0.118 |
| c.8182G>A | p.Val2728Ile | Benign | Benign | 0 | Benign | 0.002 | Tolerated | 1 |
| c.8187G>T | p.Lys2729Asn | Benign | Probably damaging | 0.998 | Probably damaging | 0.929 | Damaging | 0.001 |
| c.8308G>A | p.Ala2770Thr | Benign | Probably damaging | 1 | Probably damaging | 0.997 | Damaging | 0.001 |
| c.8503T>C | p.Ser2835Pro | Benign | Benign | 0.012 | Benign | 0.016 | Tolerated | 0.303 |
| c.8525G>A | p.Arg2842His | Benign | Probably damaging | 1 | Probably damaging | 1 | Damaging | 0 |
| c.8662C>T | p.Arg2888Cys | Benign | Possibly damaging | 0.816 | Benign | 0.103 | Tolerated | 0.103 |
| c.8734G>A | p.Ala2912Thr | Benign | Possibly damaging | 0.483 | Benign | 0.084 | Damaging | 0.016 |
| c.8764A>G | p.Ser2922Gly | Benign | Probably damaging | 0.999 | Probably damaging | 0.914 | Damaging | 0 |
| c.8830A>T | p.Ile2944Phe | Benign | Probably damaging | 0.986 | Possibly damaging | 0.809 | Damaging | 0.002 |
| c.8851G>A | p.Ala2951Thr | Benign | Probably damaging | 1 | Probably damaging | 0.999 | Damaging | 0 |
| c.8905G>A | p.Val2969Met | Benign | Possibly damaging | 0.929 | Benign | 0.107 | Damaging | 0.001 |
| c.8917C>T | p.Arg2973Cys | Benign | Probably damaging | 1 | Probably damaging | 0.92 | Damaging | 0.046 |
| c.9038C>T | p.Thr3013Ile | Benign | Possibly damaging | 0.875 | Benign | 0.208 | Damaging | 0.036 |
| c.9043A>G | p.Lys3015Glu | Benign | Benign | 0.015 | Benign | 0.004 | Tolerated | 0.086 |
| c.9175A>G | p.Lys3059Glu | Benign | Benign | 0.17 | Benign | 0.051 | Tolerated | 0.089 |
| c.9235G>A | p.Val3079Ile | Benign | Possibly damaging | 0.712 | Benign | 0.132 | Tolerated | 0.574 |
| c.9292T>C | p.Tyr3098His | Benign | Benign | 0 | Benign | 0.002 | Tolerated | 0.775 |
| c.9509A>G | p.Asp3170Gly | Benign | Benign | 0.016 | Benign | 0.039 | Tolerated | 0.124 |
| c.9592T>C | p.Cys3198Arg | Benign | Benign | 0.412 | Benign | 0.062 | Tolerated | 0.099 |
| c.9730G>A | p.Val3244Ile | Benign | Benign | 0 | Benign | 0 | Tolerated | 0.421 |
| c.9875C>T | p.Pro3292Leu | Benign | Probably damaging | 0.999 | Possibly damaging | 0.877 | Damaging | 0.001 |
| c.10045A>G | p.Thr3349Ala | Benign | Probably damaging | 1 | Probably damaging | 0.998 | Damaging | 0 |
| c.10121C>T | p.Thr3374Ile | Benign | Benign | 0.002 | Benign | 0.002 | Damaging | 0.007 |
| c.10234A>G | p.Ile3412Val | Benign | Benign | 0.002 | Benign | 0.004 | Tolerated | 0.923 |
